# Temporal Patterns and Inter-Correlations among Physical and Antioxidant Attributes and Enzyme Activities of Apricot Fruit Inoculated with *Monilinia laxa* under Salicylic Acid and Methyl Jasmonate Treatments under Shelf-Life Conditions

**DOI:** 10.3390/jof7050341

**Published:** 2021-04-28

**Authors:** Ahmed Ezzat, Szilárd Szabó, Zoltán Szabó, Attila Hegedűs, Dorina Berényi, Imre J. Holb

**Affiliations:** 1 Department of Horticulture, Faculty of Agriculture, Kafrelsheikh University, Kafr El-Shaikh 33516, Egypt; ahmed.kassem@agr.kfs.edu.eg; 2 Department of Physical Geography and Geoinformatics, University of Debrecen, H-4032 Debrecen, Hungary; szaboszilard.geo@gmail.com; 3 Faculty of Agronomy, University of Debrecen, Böszörményi út 138, 4032 Debrecen, Hungary; zoltszab@agr.unideb.hu (Z.S.); berdor@agr.deb.hu (D.B.); 4 Department of Genetics and Plant Breeding, Faculty of Horticultural Science, Szent István University, H-1118 Budapest, Hungary; hegedus.attila@uni-corvinus.hu; 5 Eötvös Loránd Research Network (ELKH), Centre for Agricultural Research, Plant Protection Institute, H-1022 Budapest, Hungary

**Keywords:** brown rot, lesion diameter, lignin, fruit firmness, phenol content, antioxidant capacity, defense related enzymes, Pearson correlation, regression analyses, PCA

## Abstract

*Monilinia laxa* causes serious postharvest damage on apricot fruits under shelf-life storage conditions. Plant elicitors of methyl jasmonate (MeJA) and salicylic acid (SA) can reduce this damage, and their research can explain the background of the plant defense physiological processes in *M. laxa*-infected fruits. The aims of this study were: (i) to evaluate the effect of various concentrations of MeJA and SA on brown rot incidence (BRI) and lesion diameter (LD) of apricot fruits; (ii) to measure the temporal patterns for the effect of 0.4 mmol L^−1^ MeJA and 2 mmol L^−1^ SA treatments on BRI, LD and seven fruit measures (fruit firmness (FF), lignin content (LC), total soluble phenol content (TSPC), total antioxidant capacity (TAC) and enzyme activities of PAL, POD and SOD) in treatments of *M. laxa*-inoculated versus (vs.) non-inoculated fruits over an eight-day shelf-life storage period; and (iii) to determine inter-correlations among the seven fruit measures for MeJA and SA treatments. Both MeJA and SA significantly reduced BRI and LD. LC, FF, TAC, TSPC, as well as SOD and PAL activities in the MeJA and SA treatments were higher than the water-treated control in most assessment days and both inoculation treatments. In both inoculation treatments, the activity of POD in the SA-treated fruits was higher than MeJA-treated and control fruits at all dates. In MeJA vs. SA and inoculated vs. non-inoculated treatments, six variable pairs (FF vs. TSPC, FF vs. TAC, TAC vs. PAL, PAL vs. POD, PAL vs. SOD, and POD vs. SOD) showed significant inter-correlation values. Principal component analyses explained 96% and 93% of the total variance for inoculated and non-inoculated treatments, respectively. In inoculated treatments, both PC1 and PC2 explained 41% of the total variance and correlated with FF, TSPC and TAC and with PAL, SOD and POD, respectively. In non-inoculated treatments, PC1 and PC2 explained 49% and 44% of the total variance and correlated with LC, PAL, POD and SOD and with FF, TSPC and TAC, respectively. It can be concluded that MeJA and SA are useful in the practice to enhance the plant defense system against brown rot by reducing fungal growth and by improving physical and antioxidant attributes (FF, LC, TAC and TSPC) and the activity of defense-related enzymes (PAL, POD and SOD) in apricot fruits during shelf-life storage conditions.

## 1. Introduction

The fungus of *Monilinia laxa* (Aderh. & Ruhl.) Honey is a serious fungal pathogen of stone fruit species, including apricot (*Prunus armeniaca* L.) [1,2,3,4,5]. The fungus can cause severe brown rot epidemics in most European stone fruit plantations [4,5,6,7,8,9,10,11]. Severe pre- and postharvest losses caused by *M. laxa* were reported in apricots [12,13,14]. Pre- and postharvest diseases, including brown rot, have been controlled successfully by chemical fungicide compounds, but due to increasing environmental concerns over chemical fungicide residues in fruits, great interest has been given to non-chemical, environmentally friendly measures for postharvest disease control [4,15,16,17,18,19].

One of the promising environmentally friendly ways for postharvest disease control is to increase the natural defense system of plants [16,19,20]. The possible prevention of fruit from fungal pathogens can be reached by the activation of the plant defense systems that help to delay the spread of various pathogens [16,21,22]. These defense mechanisms can induce plant resistance to pathogens. Such induced disease resistance in plants was shown as a possible option for preventing the spread of fungal pathogens and an attractive strategy for disease control [16,19,23,24].

A systemic acquired resistance (SAR) in plants is considered as a long-lasting immune response to pathogen infection, which relates to plant pathogen resistance [25,26]. The potential fungal disease control by SAR has been reported in the control of postharvest diseases [19,27,28,29,30,31]. Two major SAR pathways, among others, were described in plants: one involves salicylic acid (SA), and another jasmonic acid (JA) or methyl jasmonate (MeJA). These signaling compounds participate in the expression of plant resistance to pathogens, including necrotrophic pathogens such as *Monilinia* spp. [31,32,33,34,35,36,37,38,39,40].

SA is known to be an essential compound in plant pathogen resistance and participates in the expression of plant protective compounds as well as pathogenesis-related (PR) proteins and polyphenols [31,34,35,36,41,42,43,44]. Therefore, SA has been shown to be a potential compound for inhibiting postharvest fungal pathogens, and thus improves the postharvest quality of fruits. For instance, postharvest damages were effectively controlled by SA treatments in the case of *Colletotrichum gloeosporioides* on mango [45,46], *Penicillium expansum* on sweet cherry [24,34,47] and peach [48,49], *Botrytis cinerea* on peach [50], and *Monilinia* spp. on sweet cherry [34], apricot [51], apple [52] and nectarine [53].

MeJA has been demonstrated to induce SAR in plants [20,26,39,54] and participate in the biosynthesis of some plant defense compounds such as PR protein polyphenols, and alkaloids [26,38,39,54,55,56,57,58]. MeJA was shown to reduce postharvest fungal diseases of fruits [34,35,39,59,60,61], including *Monilinia* spp. in peach [35], sweet cherry [34,62], and in nectarine [5]. In a recent study [5], hormone and genetic analyses were also prepared for nectarine fruit infected with *M. laxa*, and the analyses confirmed that JA activity was likely useful for plant defense against *M. laxa*.

Only two publications made comparisons between the effect of MeJA and SA treatments in relation to fungal decay: one in sweet cherry [34] and the other in apricot [63]. In the sweet cherry publication, the effects of MeJA and SA treatments were evaluated on three defense enzymes and on brown rot incidence for the *M. fructicola*-inoculated fruits [34]. The apricot study evaluated the comparison of MeJA and SA treatments on a general fruit decay index without previous artificial inoculation, and fruit parameters during shelf-life (SL) storage conditions were measured after a preceding cold storage period [63]. However, treatments of these studies have not focused on either the comparisons of artificially inoculated versus (vs.) non-inoculated fruits or on the joint analyses of fruit quality losses, antioxidant properties, and activities of defense enzymes on diseased fruits.

Many biological connections were determined among fruit parameters for healthy fruits, which were prepared by determining the correlations among the parameters; for instance, among physical attributes, antioxidant properties, and enzyme activity [63,64,65,66,67,68]. However, there has been no attempt to present inter-correlation between fruit parameters of fruit firmness (FF), lignin content (LC), phenolic content, antioxidant capacity, and enzyme activities for treatments of *M. laxa*-inoculated vs. non-inoculated fruits under both SA and MeJA treatments, in SL storage conditions. These inter-correlations may provide additional knowledge on the background of physiological processes in plant defense mechanisms against fungal pathogens.

This work aimed, firstly, to study the effect of three MeJA and SA concentrations (0.1, 0.4 and 0.7, and 0.5, 2 and 5 mmol L^−1^, respectively) on brown rot measures (brown rot incidence (BRI) and lesion diameter (LD)) of apricot fruits; secondly, to measure the temporal patterns for effect of 0.4 mmol L^−1^ MeJA and 2 mmol L^−1^ SA treatments on BRI, LD and seven fruit measurements (physical attributes: FF, LC; antioxidant properties: total soluble phenol content (TSPC), total antioxidant capacity (TAC); and enzyme activities: PAL, POD and SOD) in treatments of *M. laxa*-inoculated vs. non-inoculated fruits during an eight-day SL storage period with assessment days 0, 2, 4, 6 and 8; and thirdly, to determine the inter-correlations (Pearson correlation, linear regression, and principal component analyses) among the above seven fruit measurements for the MeJA and SA treatments in order to understand better the physiological processes in apricot fruit in the two inoculation treatments under SL storage conditions.

## 2. Materials and Methods

### 2.1. Isolation of M. laxa

*Monilinia laxa* was isolated from diseased plum fruit. Conidia of *M. laxa* were obtained from a lesion of diseased fruit with a preparatory needle and transferred to sterile water (1 mL, with 0.5% Tween 20). The suspension was centrifuged, and then 30 µL of the suspension was evenly distributed in a Petri dish (15 × 90 mm) containing water agar (30 mL) and streptomycin sulfate (100 µg mL^−1^). Germinated *M. laxa* conidia were transferred to potato dextrose agar (PDA; WWR International, Budapest, Hungary) with a sterile preparatory needle. Germinated conidia were identified with morphological [69] and molecular diagnostic assays. Molecular diagnosis followed the procedures of Gell et al. [70] and Fazekas et al. [71] using species-specific primers. Single-spore isolates of *M. laxa* were incubated on PDA in Petri dishes under dark conditions at 20 °C, and then isolates were stored at 4 °C on PDA.

### 2.2. Plant Materials and Experimental Setup for SA and MeJA Treatments

The apricot fruit cultivar (cv.) ‘Bergarouge’ was harvested for the experiments from an integrated apricot orchard (Boldogkőváralja, Hungary). Mature fruit without visual defects were randomly selected with uniform fruit size and fruit color.

Two experimental setups were conducted: in experiment 1, three concentrations of MeJA and SA were evaluated on brown rot measures; while in experiment 2, the temporal patterns of fruit measurements were evaluated on the best concentrations of MeJA and SA under SL storage conditions.

In experiment 1, the selected fruits were classified into seven groups (60 fruits for each, 7 × 60 fruits for the seven groups) according to seven treatments (three MeJA, three SA, and one untreated control treatments). Firstly, fruit surfaces were sterilized (2% *v*/*v* sodium hypochlorite for two minutes), then fruits were cleaned with distilled water and dried. Then, fruits were immersed into a solution of 0.1, 0.4 and 0.7 mmol L^−1^ MeJA and 0.5, 2 and 5 mmol L^−1^ SA as well as into distilled water (water-treated control) for one hour. Tween 80 (0.5%, *v*/*v*) was added to the aqueous solutions of SA and MeJA, and the solutions were well-mixed to ensure homogenous suspensions. Then, fruits were divided into two further inoculation treatment groups (inoculated versus (vs.) non-inoculated, 2 × 7 × 30 fruits) for each chemical treatment. In the inoculated treatment, a wound of 4 mm in diameter was prepared in the center of each sampled fruit, and then an agar plug (3 mm in diameter) of *M. laxa* mycelia was placed into the wounds of each fruit. Then, inoculated fruits were placed into air-tight plastic boxes (60 × 40 × 15 cm) and held in SL temperature conditions (20 °C) for 8 days. In the non-inoculated treatment, fruits were placed in the boxes without inoculation. After 8 days of incubation, BRI (%) and LD (mm) were measured in each experiment. Each treatment was replicated three times (3 × 2 × 7 × 30 fruits), and the experiment was repeated twice (2 × 3 × 2 × 7 × 30 fruits). The experimental design for experiment 1 was a split plot design with the two repeated experiments as blocks, two inoculation treatments (inoculated vs. non-inoculated) as main plots, and three chemical treatments (MeJA, SA and untreated control) as sub-plots.

In experiment 2, treatments of 2 mmol L^−1^ SA, 0.4 mmol L^−1^ MeJA (the most appropriate concentrations resulting from experiment 1) and water-treated control were prepared as described for experiment 1. The used numbers of fruits were: 3 treatment groups × 2 inoculation treatments × 3 treatment replications × 2 experiment repetitions × 30 sample fruits. In this experiment, measurements for BRI, LD, physical attributes, antioxidant properties, and the activity of defense-related enzymes were assessed at 0, 2, 4, 6 and 8 days during the SL storage conditions. The experimental design for experiment 2 was a split-split plot design with the two repeated experiments as blocks, two inoculation treatments (inoculated vs. non-inoculated) as main plots, three chemical treatments (MeJA, SA and untreated control) as sub-plots, and five assessment dates (days 0, 2, 4, 6 and 8) as sub-sub plots.

### 2.3. Brown Rot Measures: Brown Rot Incidence and Lesion Diameter

Both BRI and LD were assessed at day 8 and at 0, 2, 4, 6, and 8 days after the treatment began in experiments 1 and 2, respectively. Fruits were considered as brown rot-diseased if at least a 1 mm brown rot symptom or a black stroma formation had appeared on the fruit. Then, the percentage of BRI was computed as BRI (%) = (number of diseased fruit/total number of assessed fruit) × 100. Brown rot LD was measured with a digital caliper.

### 2.4. Physical Attributes: Fruit Firmness and Lignin Content

Fruit firmness (FF, kg cm^−2^) was determined at 0, 2, 4, 6 and 8 days after the treatment began in experiment 2, using a penetrometer (Magness Tazlor type—model FT011, Florence, Italy).

A gravimetric method [72] was used to determine lignin content (LC) at 0, 2, 4, 6 and 8 days after the treatment began for experiment 2. Fruit samples were dispersed into a solution of H_2_SO_4_ (72%, room temperature, 6 h), which was then diluted to 1 M H_2_SO_4_ and boiled at 100 °C for 2.5 h. After filtering the solution, the remaining insoluble material was washed with hot water at 90 °C until the sample become acid-free. Then, a drying procedure (at 105 °C for overnight) was applied to the sample. The weight of the dried residue was considered as LC, expressed as a percentage of fresh weight (%FW).

### 2.5. Antioxidant Properties: Total Soluble Phenol Content and Total Antioxidant Capacity

The amount of total soluble phenol content (TSPC) of fruits was measured using Folin–Ciocalteu (FC) reagent [73] in experiment 2, at 0, 2, 4, 6 and 8 days after treatment began. Following the FC protocol of the study of Singleton and Rossi [72], the determination of TSPC amounts was based on a standard calibration curve gained from various concentrations of gallic acid (GA). Then, TSPC was expressed as GA equivalents (GAEs) in mg for a 100 g fresh weight sample (GAE 100 g^−1^ FW).

Total antioxidant capacity (TAC) of fruits was measured spectrophotometrically with the method of ferric reducing antioxidant power (FRAP) [73] in experiment 2, at 0, 2, 4, 6 and 8 days after treatment began. Following the FRAP protocol of the study of Benzie and Strain [74], TAC measurement was based on the Fe^3+^-TPTZ (ferric-tripyridyltriazine) complex changes to [Fe^2+^-TPTZ] at low pH (pH 3.6). Then, the measure for absorption change of Fe^2+^-TPTZ was prepared at 593 nm wavelength. Results were expressed as ascorbic acid (AA) equivalents in mg for 1 g FW (mg AA g^−1^ FW).

### 2.6. Activity of Defense-Related Enzymes

Activity of defense-related enzymes in fruits was sampled in experiment 2, at 0, 2, 4, 6 and 8 days after the treatment began.

For PAL activity, fruit flesh (10 g) from 10 fruits were prepared, and then the sample was homogenized with sodium borate buffer (SBB) and polyvinyl pyrrolidone (PVP): 25 mL of 50 mmol L^−1^ SBB containing 5 mmol β-mercaptoethanol at pH 8.8 and 0.5 g PVP were used. Then, the activity of the enzyme was determined using the slightly modified method of Assis et al. [75]. Briefly, 1 mL enzyme extract and 2 mL of borate buffer (50 mmol L^−1^, pH 8.8), together with 1 mL of L-phenylalanine (20 mmol L^−1^), was incubated in a climate chamber at 37 °C. After 60 min incubation, 1 mL HCl (1 mol L^−1^) was added to the enzyme extract in order to stop the reaction. The trans-cinnamic acid (T-CC) production was measured for determining the activity of the enzyme. T-CC was then measured spectrophotometrically at 290 nm wavelength. The mixture of a crude enzyme preparation and L-phenylalanine without incubation was used as a blank. Enzyme activity of PAL was then expressed as nmol CC h^−1^ mg^−1^ protein.

For SOD activity, fruit tissue (1 g) was frozen, then ground in 5 mL sodium phosphate buffer (50 mmol L^−1^) at pH 7.0. The extracts were then homogenized and centrifuged (10,000× *g*, 20 min, 4 °C). The supernatants remaining after centrifugation were used to determine enzyme activity. Enzyme activity was then determined photochemically, following the methodological procedure of Rao et al. [76]. Then, a reaction mixture (3 mL) was prepared containing EDTA (3 µmol L^−1^), sodium phosphate (50 mmol L^−1^, pH 7.8), nitro-blue-tetrazolium (NBT; 1 µmol L^−1^), methionine (14 mmol L^−1^), riboflavin (60 µmol L^−1^), and crude enzyme extract (0.1 mL). In the reaction mixture, absorbance of blue formazan was measured spectrophotometrically at 560 nm wavelength. Enzyme activity for one unit (1 U) was determined based on the 50% inhibition capacity of the enzyme to NBT. Then, enzyme activity of SOD in fruit was expressed as one U mg per protein (U mg^−1^ FW min^−1^).

For POD activity, a mixture of apricot fruit tissue sample (1 g) and sodium phosphate buffer (5 mL, 0.2 mol L^−1^, pH 8.7) was ground; the mixture was homogenized then centrifuged (10,000× *g*, 20 min, 4 °C). In the remaining supernatants, POD was determined with a modified protocol of the study of Kochba et al. [77]. Then, a reaction mixture (3 mL) was prepared containing sodium acetate (50 mM, pH 5.4), guaiacol (20 mM), H_2_O_2_ (0.75%) and crude enzyme extract (0.2 mL). Enzyme activity was measured spectrophotometrically by determining the absorbance. In the mixture, the increase in the absorbance at 460 nm wavelength was determined, and POD activity of one unit (U) was defined as the change in absorbance in 0.01 units for 1 min. Bovine serum albumin was used as a standard for estimating the protein content of the enzyme extracts. POD enzyme activity was expressed as U per milligrams of protein (U mg^−1^ FW min^−1^).

### 2.7. Statistical Analysis

#### 2.7.1. ANOVA

In experiment 1, a split plot analysis of variance was used for BRI and LD separately for the inoculation and chemical treatments of fruit datasets. The effects of inoculation treatment (inoculated vs. non-inoculated), chemical treatment (MeJA, SA, and untreated control), and their interactions were determined on the two measurements.

In experiment 2, mean data of the sub-sub plots were used for the statistical analyses in order to obtain a single value for each measurement. Then, split–split plot ANOVA was prepared to analyze the measurements separately for the two inoculation and the three chemical treatments as well as for the five assessment dates of fruit datasets. The effects of inoculation treatment (inoculated vs. non-inoculated), chemical treatment (MeJA, SA, and untreated control), assessment date (days 0, 2, 4, 6 and 8), and their interactions were evaluated on the measurements.

Collected datasets were analyzed in the SPSS program (SPSS Inc., Chicago, IL, USA) in both experiments. Before the analyses, BRI values were arcsine-square-root-transformed and an LSD_0.05_ *t*-test was used at *p* = 0.05 level for treatment separation.

#### 2.7.2. Correlation and Regression Analysis among Measurements

In order to determine the pair-wise relationship among the seven fruit measurements (FF, LC, TSPC, TAC, PAL, POD and SOD), Pearson’s correlation coefficients were quantified for the relationships among the seven fruit measurements in all combinations. Correlation coefficients and their corresponding significance levels (at *p* = 0.05 probability level) were determined separately for the inoculation–chemical treatment combinations (inoculation vs. MeJA, inoculation vs. SA, non-inoculation vs. MeJA, and non-inoculation vs. SA). Genstat 5 Release 4.1 (Lawes Agricultural Trust, Rothamsted, UK) was used for the analyses. Then, the best-correlated pair-wise variables were further analyzed. The strongest pair-wise relationships among the seven fruit measurements were further analyzed by linear regression analyses (f(x) = ax + b). Then, a *t*-test was used for comparing regression slopes in order to express the differences between MeJA and SA treatments separately for inoculated and non-inoculated fruits at *p* = 0.05.

#### 2.7.3. Principal Component Analysis

The seven variables (FF, LC, TSPC, TAC, PAL, POD and SOD) were further analyzed by a standardized PCA (principal component analysis) applied with a Varimax rotation. PCA was performed separately for inoculated vs. non-inoculated treatments. The values of all the seven variables were standardized by transforming them to z-scores. Root mean square residuals (RMSRs) were used to test model fit [78]. Biplot diagrams were prepared for visualizing principal components (PCs). PCA was performed by using R 4.03 [79] with the psych [80], FactoMiner [81] and factoextra [82] packages (R Core Team, Vienna, Austria).

## 3. Results

### 3.1. Experiment 1: Brown Rot Incidence and Lesion Diameter in Three Concentrations of MeJA and SA Treatments

Data for BRI and LD are not shown for non-inoculated treatments, because brown rot did not appear. ANOVA of BRI and LD demonstrated significant (*p* < 0.05) differences for the chemical treatments (Table 1).

All concentrations of MeJA and SA decreased BRI on fruit at *p* = 0.05 more than the water-treated control fruit at day 8 after the treatment began (Table 1). Treatments of 0.4 mmol L^−1^ MeJA and 2 mmol L^−1^ SA showed the largest reductions in BRI, which were significantly different from 0.1 mmol L^−1^ MeJA and 0.5 mmol L^−1^ SA treatments, respectively, but were not significantly different from 0.7 mmol L^−1^ MeJA and 5 mmol L^−1^ SA treatments, respectively (Table 1).

Similarly to BRI, LD on fruit decreased with all SA and MeJA treatments at *p* = 0.05 more than control fruits at day 8 after treatment began (Table 1), except for the treatment of 0.1 mmol L^−1^ MeJA. Treatments of 0.4 mmol L^−1^ MeJA and 2 mmol L^−1^ SA showed the largest reductions in LD, which were significantly different from 0.1 and 0.7 mmol L^−1^ MeJA and 0.5 mmol L^−1^ SA treatments, respectively, although the 2 mmol L^−1^ SA treatment was not significantly different from the 5 mmol L^−1^ SA treatment (Table 1).

According to the results, 0.4 mmol L^−1^ MeJA and 2 mmol L^−1^ SA showed the highest reduction with the lowest concentration in both BRI and LD; therefore, only these treatments were retained for further temporal pattern studies in experiment 2.

### 3.2. Experiment 2: Temporal Patterns of Brown Rot Measures

ANOVA of BRI and LD demonstrated significant (*p* < 0.05) differences for the chemical treatments and assessment dates. Significant two-way interactions were not detected amongst treatment factors. Therefore, data were presented separately for chemical treatments and assessment dates.

**Figure 1:**
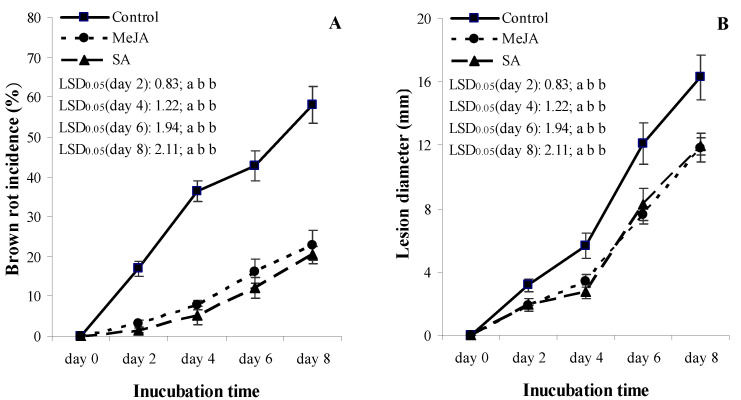
Effect of 2 mmol L^−1^ salicylic acid (SA) and 0.4 mmol L^−1^ methyl jasmonate (MeJA) on brown rot incidence (%) (**A**) and on lesion diameter (mm) (**B**) of apricot fruit (cv. ‘Bergarouge’) inoculated with *Monilinia laxa* assessed at days 0, 2, 4, 6 and 8 after treatment began. Standard deviation values are presented by bars. Control refers to fruits treated with distilled water. Differences among the control, MeJA, and SA treatments are represented by LSD_0.05_ values at *p* < 0.05. Values within the given days coupled with different letters are significantly different among the water-treated control, MeJA, and SA treatments at *p* = 0.05 according to LSD *t*-tests. After each LSD_0.05_ value, the first, second, and third letters belong to control, MeJA, and, SA treatments, respectively. Fruits showed no brown rot symptoms in non-inoculated treatments; therefore, these data were omitted.

Again, both SA and MeJA treatments decreased BRI and LD on fruit inoculated with *M. laxa* at *p* = 0.05 compared to the water-treated control, on all assessment dates except from day 0 after the treatment began (Figure 1A,B). Values of both BRI and LD for the SA treatment were not different from the corresponding MeJA treatments at any of the assessment days at *p* = 0.05. Fruits showed no brown rot symptoms in the non-inoculated treatment groups.

### 3.3. Experiment 2: Temporal Patterns of Fruit Firmness and Lignin Content

ANOVA of FF and LC indicated differences amongst inoculation treatments, chemical treatments, and assessment dates at *p* < 0.05. No significant two- and three-way interactions were detected amongst treatment factors. Therefore, data are presented separately for inoculation treatments, chemical treatments, and assessment dates.

**Figure 2:**
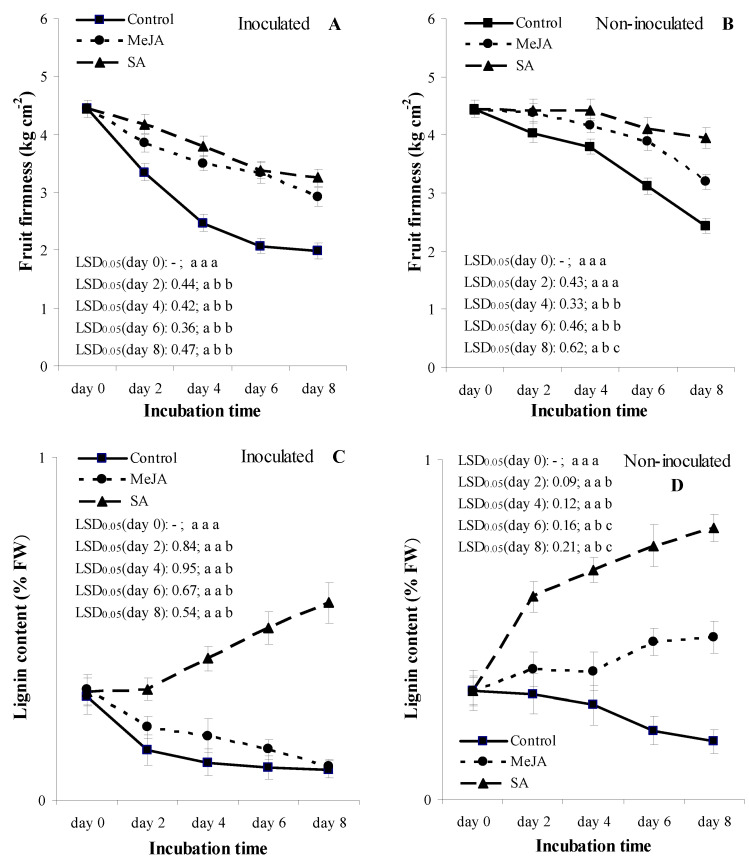
Effect of 2 mmol L^−1^ salicylic acid (SA) and 0.4 mmol L^−1^ methyl jasmonate (MeJA) on fruit firmness (kg cm^−2^) (**A**,**B**) and on lignin content (% FW) (**C**,**D**) of apricot fruit (cultivar ‘Bergarouge’) in treatments of inoculated with *Monilinia laxa* (**A**,**C**) and non-inoculated (**B**,**D**) fruits assessed at days 0, 2, 4, 6 and 8 after treatment began. Information on symbols, error bars and letters for LSD_0.05_ values is presented in Figure 1.

In the inoculated treatments, values of FF for the MeJA and SA treatments were generally lower than the corresponding non-inoculated treatments (Figure 2A,B). In the inoculated treatments, FF of fruits continuously decreased in all the three treatments from assessment days 0 to 8. FF values of MeJA and SA treatments were significantly higher at *p* = 0.05 than the water-treated control from assessments days 2 to 8, but corresponding MeJA and SA treatments did not differ from each other during the same assessment periods (Figure 2A).

In the non-inoculated treatments, values of FF for the MeJA and SA treatments were significantly higher (*p* = 0.05) compared to the water-treated fruits from assessment days 4 to 8 (Figure 2B). SA-treated fruits were generally firmer compared to MeJA-treated fruits, but the differences between SA and MeJA treatments were only significant at *p* = 0.05 at assessment day 8.

In the inoculated treatments, the LC of fruit was generally lower in all treatments compared to the non-inoculated treatments (Figure 2C,D). A significant increase in LC was detected in the SA treatments in all assessment dates when compared with either the water-treated control or the MeJA-treated fruits (Figure 2C). LC of fruit was not significantly different between water-treated and the MeJA-treated fruits on any assessment date.

In the non-inoculated treatments, the LC of fruits was higher at *p* = 0.05 in the treatments of SA and MeJA than that of control fruit at the assessment days 2 to 8, and at the assessment days 6 and 8, respectively (Figure 2D). LC of fruits was the highest in the SA treatment, where LC increased at all assessment days. LC of fruit was higher at *p* = 0.05 in the SA treatments compared with the corresponding MeJA treatments from assessment days 2 to 8.

### 3.4. Experiment 2: Temporal Patterns of Total Soluble Phenol Content and Total Antioxidant Capacity

Analyses of variance of TSPC and TAC demonstrated differences at *p* < 0.05 amongst inoculation treatments, chemical treatments, and assessment dates. No significant two- and three-way interactions were detected amongst treatment factors (*data not shown*). Therefore, data are presented separately for inoculation treatments, chemical treatments, and assessment dates.

**Figure 3:**
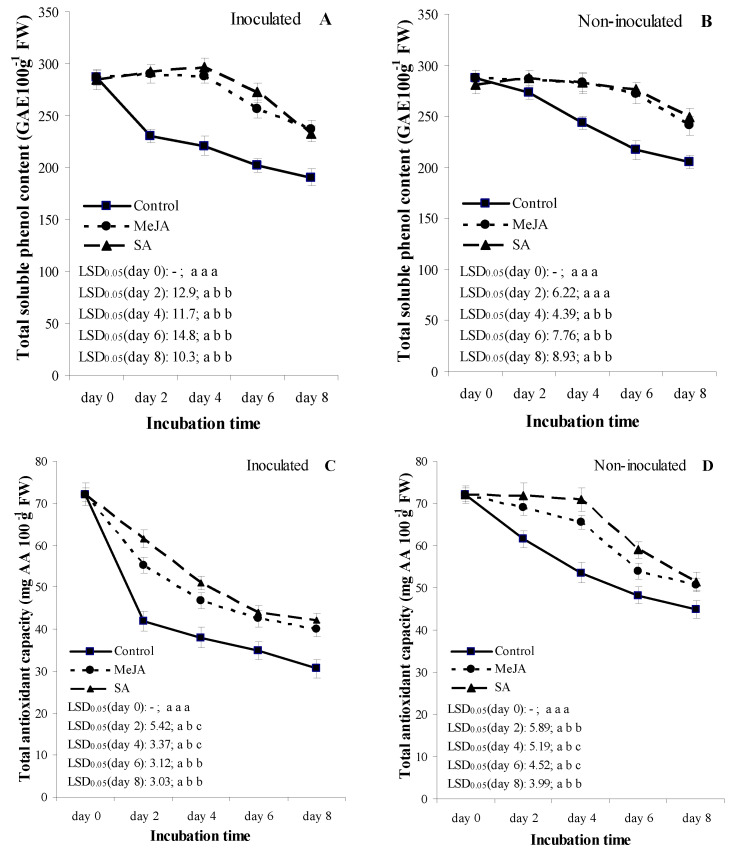
Effect of 2 mmol L^−1^ salicylic acid (SA) and 0.4 mmol L^−1^ methyl jasmonate (MeJA) on total soluble phenol content (GAE 100 g^−1^ FW) (**A**,**B**) and on total antioxidant capacity (mg AA 100 g^−1^ FW) (**C**,**D**) in apricot fruit (cultivar ‘Bergarouge’) in treatments inoculated with *Monilinia laxa* (**A**,**C**) and non-inoculated (**B**,**D**) fruits assessed at days 0, 2, 4, 6 and 8 after treatment began. Information on symbols, error bars and letters for LSD_0.05_ values is presented in Figure 1.

In the inoculated treatments, TSPC of fruit for the MeJA and SA treatments showed similar temporal patterns as in the non-inoculated treatments (Figure 3A,B). TSPC increased in the inoculated treatments for both compounds until day 4, then decreased until the final assessment day (Figure 3A). The TSPC of the control fruits decreased from assessment day 0 to day 8, and their values were significantly lower compared to either MeJA or SA treatments at each assessment date. Values of the TSPC were not significantly different between the MeJA and SA treatments (Figure 3A).

In the non-inoculated treatments, the TSPC of fruits with the MeJA and SA treatments were higher at *p* = 0.05 than the water-treated control treatments from assessments days 4 to 8 (Figure 3B). Values of the TSPC were not significantly different between the MeJA- and SA-treated fruits at any assessment days.

In the inoculated treatments, temporal pattern of TAC showed a continuous decrease in all treatments until the final assessment day (Figure 3C, and the values were lower on each assessment date compared to the corresponding values of the non-inoculated treatments (Figure 3C,D). Values of TAC in the MeJA and SA treatments were significantly higher than those of the water-treated control fruit from assessment days 2 to 8 (Figure 3D). SA-treated fruits showed higher TAC at *p* = 0.05 compared with MeJA-treated fruits at assessment days 2 and 4.

In the non-inoculated treatments, TAC of fruits was higher at *p* = 0.05 in the MeJA and SA treatments compared with the water-treated control fruit from assessment days 2 to 8 (Figure 3D). Values of SA-treated fruits showed higher TAC at *p* = 0.05 than that of MeJA-treated fruits at assessment days 4 and 6.

### 3.5. Experiment 2: Temporal Patterns of the Activity of Defense-Related Enzymes

ANOVA of PAL, POD and SOD activities demonstrated differences amongst inoculation and chemical treatments as well as amongst assessment dates at *p* < 0.05. No significant two- and three-way interactions were detected amongst treatment factors. Therefore, data are shown separately for all treatments and assessment dates.

**Figure 4:**
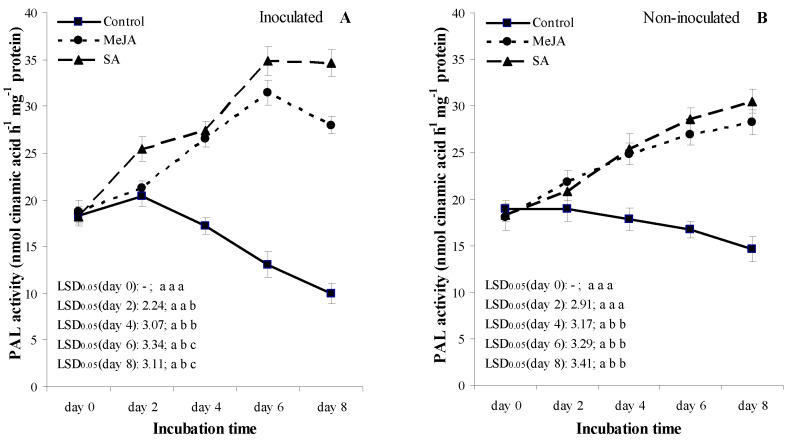

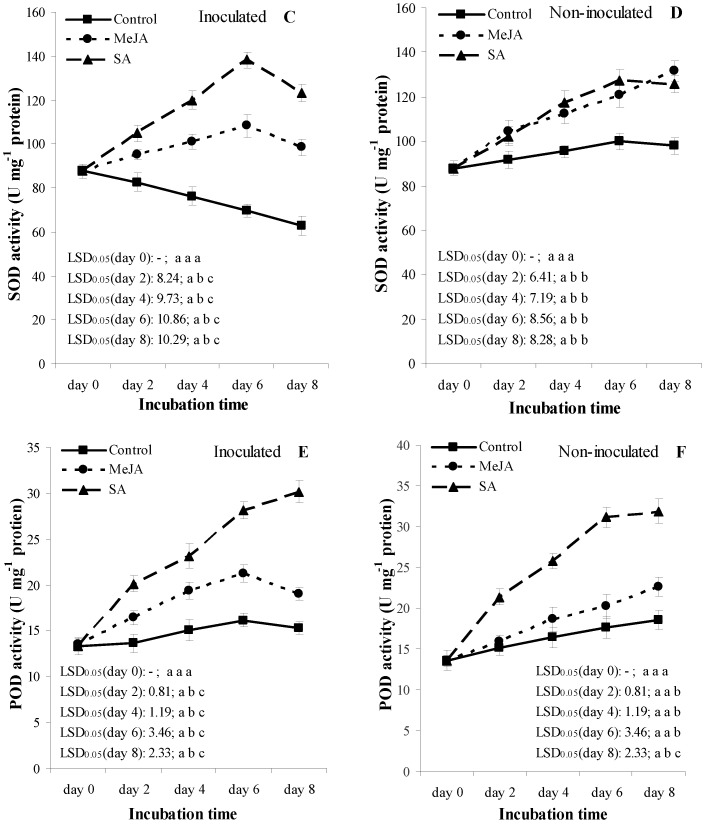
Effect of 2 mmol L^−1^ salicylic acid (SA) and 0.4 mmol L^−1^ methyl jasmonate (MeJA) on phenylalanine ammonia-lyase (PAL; nmol cinnamic acid h^−1^ mg^−1^ protein) activity (**A**,**B**), on superoxide dismutase (SOD; U mg^−1^ protein) activity (**C**,**D**) and on peroxidase (POD; U mg^−1^ protein) activity (**E**,**F**) in apricot fruit (cultivar ‘Bergarouge’) in treatments inoculated against *Monilinia laxa* (**A**,**C**) and non-inoculated (**B**,**D**) fruits assessed at days 0, 2, 4, 6 and 8 after treatment began. Information on symbols, error bars and letters for LSD_0.05_ values is presented in Figure 1.

In the inoculated treatments, an increase in PAL activity was detected in MeJA and SA treatments until assessment day 6 (Figure 4A). PAL activity of SA and MeJA treatments were significantly different (*p* = 0.05) from the water-treated control at assessment days 2, 4, 6 and 8, and assessment days 4, 6 and 8, respectively (Figure 4A). PAL activity in fruits was higher in the SA treatments at *p* = 0.05 compared with the MeJA treatments at assessment days 2, 6 and 8. A considerable decrease in PAL activity was detected in the water-treated control treatments after assessment day 2 until the final assessment day (Figure 4A).

In the non-inoculated treatments, temporal patterns of PAL activity in fruits showed a continuous increase in MeJA and SA treatments, while a decrease was found in the water-treated control fruits (Figure 4B). PAL activity in fruits was non-significant between the MeJA and SA treatments at all assessment days, but the PAL activity in fruit was higher in both treatments at *p* = 0.05 compared to the water-treated fruits at the assessment period between days 4 and 8.

Trends of temporal patterns for SOD activity were similar to values of PAL activity in both inoculation treatments (Figure 4A–D). The temporal pattern differences between PAL and SOD activities were as follows: (i) SOD activity of fruits in the inoculated treatments was higher in the SA treatments at *p* = 0.05 compared with the MeJA treatments at the assessment period between day 2 and 8; (ii) an increase in the temporal patterns of SOD enzyme activity was observed in the water-treated control treatments for the non-inoculated fruits; and (iii) SOD activity in fruit was higher in both treatments at *p* = 0.05 compared with the water-treated fruits at the assessment period between days 2 and 8 in the non-inoculated treatments (Figure 4C,D).

In both inoculation treatments, POD activity in fruits continuously increased in all treatments from the early assessment days until day 6 in the order of water-treated control, MeJA, and SA treatments (Figure 4E,F). Temporal patterns of SA treatments demonstrated the highest POD activity in both inoculation treatments, which were significantly different from the corresponding MeJA and water-treated control treatments at the period from day 2 to 8. Values of MeJA treatments were significantly different from the corresponding values of the control treatments in the inoculated and non-inoculated treatments in the period between days 2 and 8 and at day 8, respectively.

### 3.6. Correlation and Regression Analysis among Measurements

When 4 × 21 parameter pairs were subjected to Pearson’s correlation analyses, 12, 14, 13, and 10 parameter pairs correlated significantly at *p* < 0.05 in inoculated MeJA, inoculated SA, non-inoculated MeJA, and non-inoculated SA treatments, respectively (Table 2 and Table 3). Among these, six parameter pairs were significant in both MeJA and both SA treatments. The five parameter pairs (FF vs. TSPC, FF vs. TAC, PAL vs. POD, PAL vs. SOD, and POD vs. SOD) were correlated positively, and one pair-variable (TAC vs. PAL) was correlated negatively (Table 2 and Table 3).

**Figure 5:**
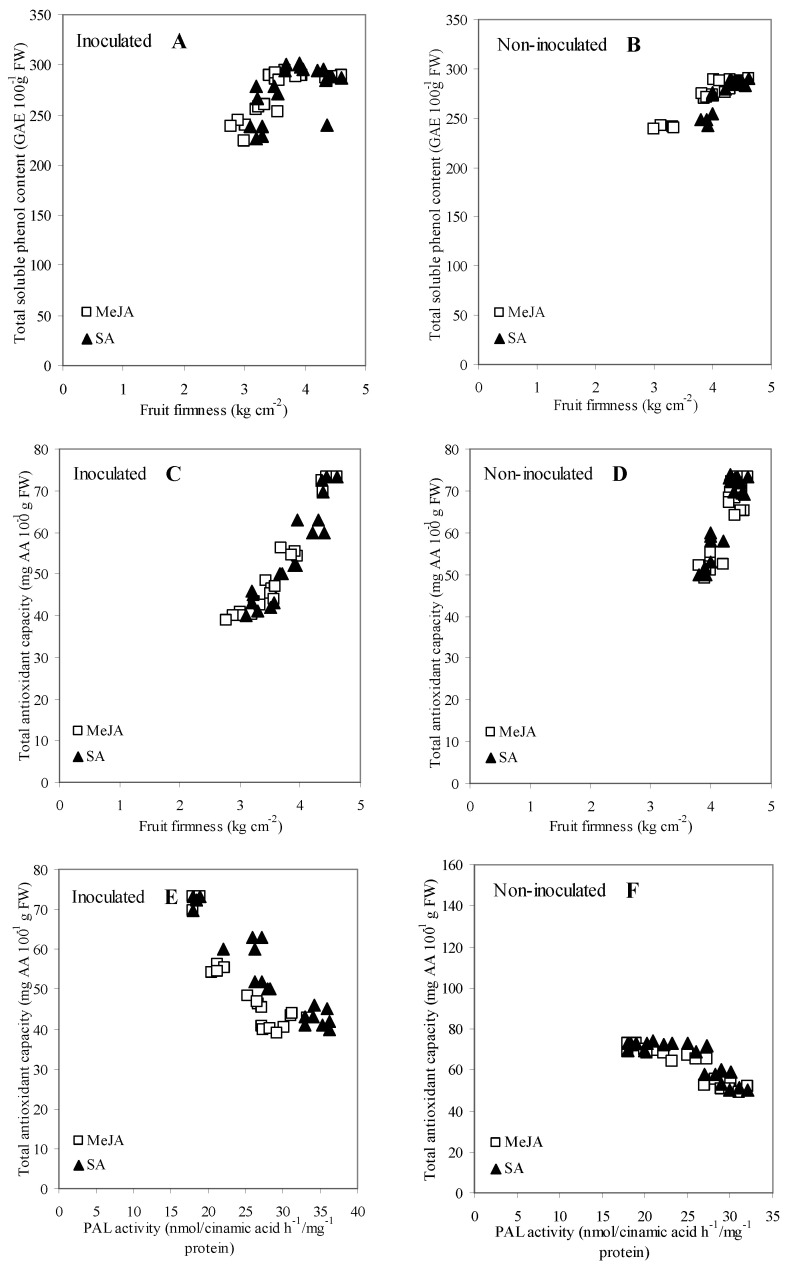

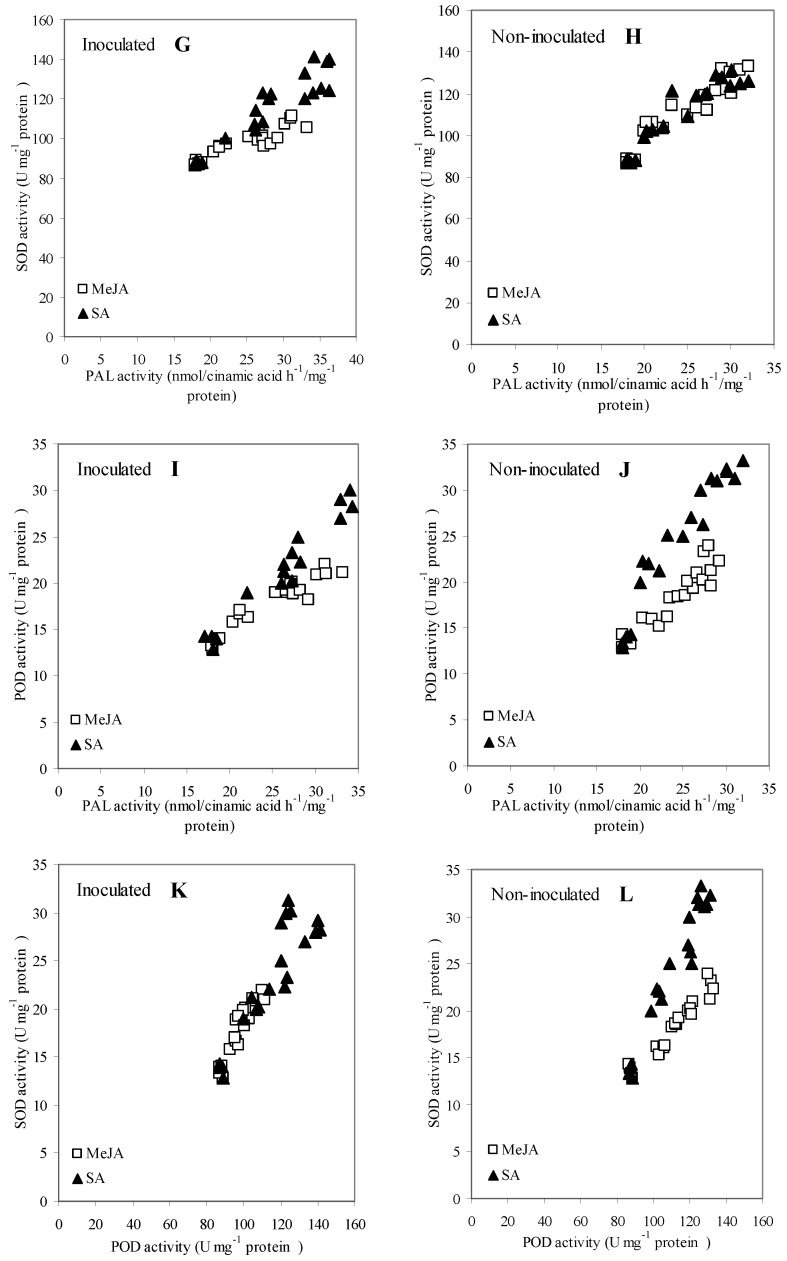
Relationships between fruit firmness (FF) and total soluble phenol content (TSPC) (**A**,**B**), FF and total antioxidant capacity (TAC) (**C**,**D**), TAC and phenylalanine ammonia-lyase (PAL) activity (**E**,**F**), PAL activity and peroxidase (POD) activity (**G**,**H**), PAL activity and superoxide dismutase (SOD) activity (**I**,**J**), POD activity and SOD activity (**K**,**L**) in treatments with methyl jasmonate (MeJA, 0.4 mmol L^−1^) and salicylic acid (SA, 2 mmol L^−1^) separately for inoculated (**A**,**C**,**E**,**G**,**I**,**K**) and non-inoculated treatments (**B**,**D**,**F**,**H**,**J**,**L**) on cultivar ‘Bergarouge’ apricot fruit.

The relationships of these six parameter pairs were further demonstrated by linear regression analysis separately for inoculated and non-inoculated treatments (Figure 5). This analysis revealed significant linear relationships for all the six parameter pairs with *r* = 0.721–0.914, *p* = 0.04–0.001, and with *r* = 0.701–0.897, *p* = 0.04–0.001 for both MeJA and both SA treatments, respectively. However, no differences were observed among the slope parameters for all pair-variables in either the inoculated or the non-inoculated treatments, between the MeJA and the SA treatments (*t*-tests showed *p* = 0.765–0.123).

### 3.7. Principal Component Analysis among Measurements

**Figure 6:**
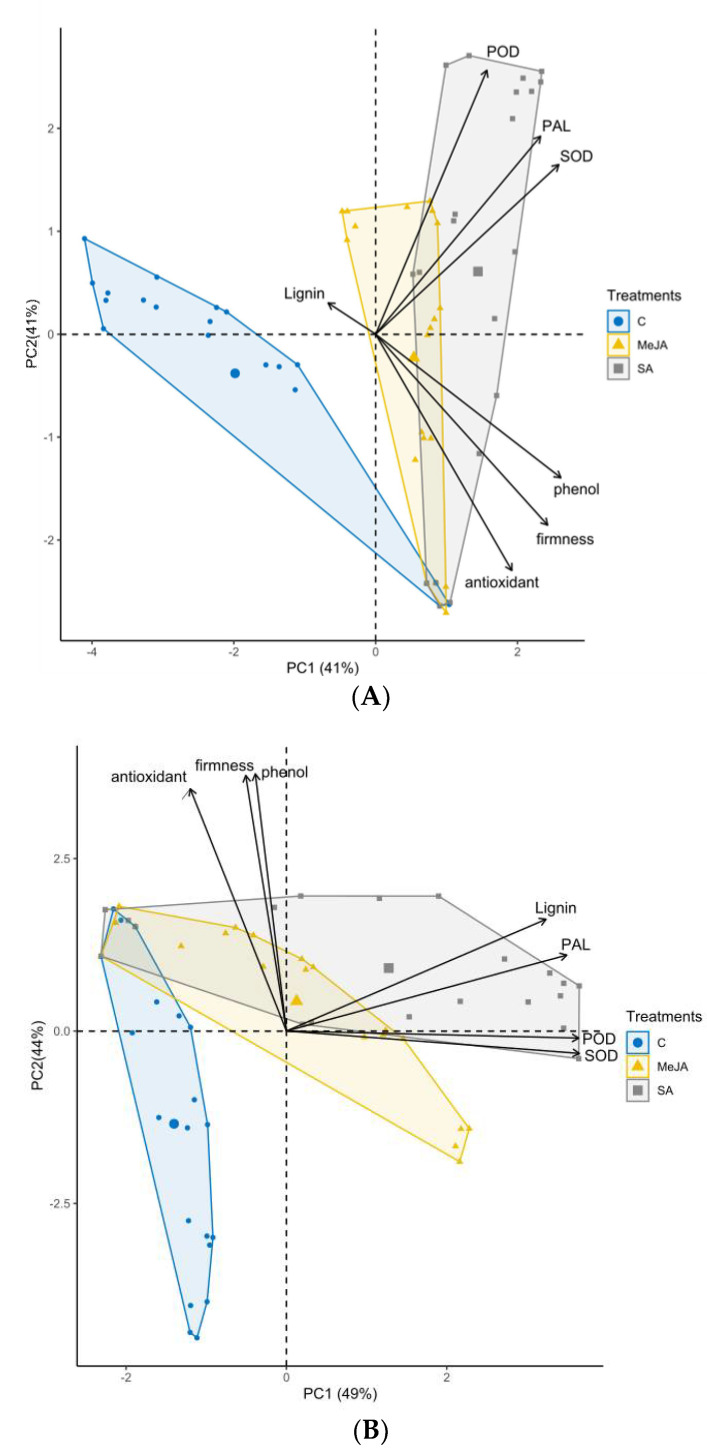
Two biplots of PCA (principal component analyses) conducted separately for *Monilinia laxa*-inoculated (**A**) and non-inoculated (**B**) apricot fruits (cultivar ‘Bergarouge’) on the measured fruit quality parameters: FF, fruit firmness; LC, lignin content; TSPC, total soluble phenol content; TAC, total antioxidant capacity; PAL, phenylalanine ammonia-lyase activity; SOD, superoxide dismutase activity; and POD, peroxidase activity. C: control; MeJA: MeJA, 0.4 mmol L^−1^; SA: SA, 2 mmol L^−1^; 95% ellipses.

PCA explained 96% and 93% of the total variance for inoculated and non-inoculated treatments, respectively (Figure 6). The number of PCs had been justified by the RMSR: 0.02 for the inoculated (three PCs) and 0.04 (two PCs) for the non-inoculated treatments, indicating a very good fit.

In the inoculated treatments, both PC1 and PC2 explained 41% of the total variance and correlated with the FF, TAC and TSPC, and with the PAL, SOD and POD variables, respectively (Figure 6A). In addition, 14% of the variance was accounted for by PC3 and correlated only with the LC.

The biplot figure shows that both PC1 and PC2 axes were dominant for all three treatments (control, MeJA, SA), but treatments were separated by the physical and antioxidant variables of PC1 (FF, TAC and TSPC) and defense enzyme variables of PC2 (PAL, SOD and POD). The decisive role of these two groups was also indicated by the arrows’ length and proximity within the PC1 variable group of physical and antioxidant properties (FF, TAC and TSPC) and within the PC2 variable group of defense enzymes (PAL, SOD and POD). All these indicated strong physiological associations among physical, antioxidant, and enzyme activity properties (Figure 6A).

In the non-inoculated treatments, PC1 and PC2 accounted for 49% and 44% of the variance and correlated with the LC, PAL, POD and SOD, and with the FF, TAC and TSPC variables, respectively (Figure 6B). The biplot figure shows that PC2 axes were dominant for the control, but MeJA and SA treatments separated variables mainly throughout the PC1 variables (LC, PAL, POD and SOD), although the decisive role of PC2 variables (FF, TAC and TSPC) were considerable too. Again, the arrows’ length and proximity of the included variables within the two PC groups indicated strong physiological associations among physical, antioxidant, and enzyme activity properties in the *M. laxa*-infected fruits (Figure 6B).

## 4. Discussion

In this study, postharvest treatments of SA (0.5, 2 and 5 mmol L^−1^) and MeJA (0.4 and 0.7 mmol L^−1^) significantly reduced the BRI or LD of apricot fruit in an eight-day assessment period during SL storage conditions (Table 1; Figure 1). Our results support the previous reports of Yao and Tian [34] on *M. fructicola* vs. sweet cherry, and of Cao et al. [59] on *Colletotrichum acutatum* vs. loquat pathosystems. The results of Yao and Tian [34] indicated that pre-harvest treatment with SA or MeJA could significantly reduce disease incidence of sweet cherry fruit stored at 25 °C; however, postharvest treatment with SA or MeJA did not reduce the disease incidence of fruit following inoculation with *M. fructicola*. Our results may be due to a direct toxicity effect of these elicitors on *M. laxa* mycelia and/or to an indirect plant defense-related effect by inducing resistance in the infected cells. Tsao and Zhou [62] showed that 500 µg mL^−1^ MeJA reduced sweet cherry brown rot (*M. fructicola*) only in a mixture with 500 µg mL^−1^ carvacol, concluding that MeJA had no direct antifungal activity and only elicits the induction of phytoalexins against postharvest diseases. On the other hand, Yao and Tian [34] reported that 2 mmol L^−1^ SA significantly inhibited hyphal growth and conidia germination of *M. fructicola* in vitro, showing a direct toxicity on the fungus of *M. fructicola*. The same authors also showed that pre-harvest applications of SA or/and MeJA reduced BRI and LD at *p* = 0.05 on sweet cherry fruit compared with non-treated fruit. Yao and Tian [34] concluded that the roles of SA or MeJA in reducing brown rot may be due to the direct toxicity of both compounds on fungal mycelia and/or to an indirect plant defense-related effect by the activation of some defense enzymes which have an important role, including: (i) to break down the cell wall of the fungus (such as chitinase and β-1,3-glucanase); (ii) saving the plant cell wall; or (iii) raising the antioxidant capacity in the cells (such as PAL or POD). In addition, Cao et al. [59] stated that MeJA controls *C. acutatum* directly by the inhibition of the pathogen growth on loquat fruits, and indirectly by inducing disease resistance triggered by an enhanced H_2_O_2_ level. In summary, our results on disease reduction in the *M. laxa* vs. apricot pathosystem may also be explained by the above mechanisms, i.e., direct or indirect fungal toxicity, saving plant cell wall by increasing lignin content, and raising defense enzyme activity in the cells (such as PAL, SOD or POD), which were also shown in treatments of 2 mmol L^−1^ SA and/or 0.4 mmol L^−1^ MeJA under SL storage conditions (Table 1, Figure 1, Figure 2 and Figure 4).

The ability of SA and MeJA to improve the resistance of apricot fruit to *M. laxa* infection was accompanied with an ability to preserve fruit firmness, even after infection, much higher than the water-treated control treatment (Figure 2A,B). This higher firmness may be why SA and MeJA are known as ethylene biosynthesis inhibitors; thus, they reduce fruit softening and delay over-ripening [83,84,85]. Babalar et al. [86] showed that SA delayed the maturity of strawberry fruit, explained by the inhibition of ethylene biosynthesis. In addition, Asghari and Aghdam [43] demonstrated that SA inhibited membrane- and cell wall-degrading enzymes, such as lipoxygenase, polygalacturonase, cellulase and pectin methyl-esterase, leading to a decreased fruit softening rate. In the *M. laxa*-inoculated fruits, our results also revealed that FF of SA and MeJA treatments had strong relationships with antioxidant properties (TAC and TSPC) in all the three inter-correlation analyses (Pearson correlation, regression analyses, and PCA) (Table 2 and Table 3, Figure 5 and Figure 6). These results indicated that the increasing TAC and TSPC, induced by MeJA and SA, were able to maintain the firmness of infected fruits, and a successful delay in the growth of *M. laxa* (BRI and LD) was detected in the infected fruits (Figure 1).

Lignin is a complex compound of phenylpropanoid, which mainly belongs to cell walls [87,88], and lignin synthesis can be induced by either mechanical wounding or microorganisms [89,90,91]. For the final step of lignin biosynthesis, there is a need for oxygen in order to oxidate the manomeric lignin precursors (e.g., coniferyl, p-courmaryl and sinapyl alcohols). The oxidation occurs through the action of peroxidase and results in lignin polymers [86,91]. In our study, the stable fruit firmness of apricot fruit may have been due to a more rapid accumulation of lignin in MeJA- or SA-treated fruit (Figure 2) than in the control. The increase in TSPC (Figure 3) was in a strong association with high activities of PAL and POD (Figure 4), which play essential roles in the phenylpropanoid pathway [49,92]. This result suggested that SA or MeJA was able to prevent fruit from softening by influencing the enzymes of lignin biosynthesis such as PAL and POD. Su et al. [93] also noted that MeJA treatment increased the activities of PAL and POD and LC at *p* = 0.05, which can enhance disease resistance and decrease the disease level in MeJA-treated vegetable soybean. These findings were in line with the results of this study; we also reported an increase in PAL and POD activities (Figure 4) with lower disease incidence (Figure 1), as well as with a higher LC (Figure 2) in the cell wall of apricot, which were associated with the *M. laxa*-inoculated fruit treated with MeJA.

Phenol substances are naturally occurring plant compounds, which play the main physiological roles in the reduction in fungal plant diseases, and plant phenolics are scavengers of several oxidizing molecules [94,95]. Phenols in plants are synthesized through the shikimate–phenylpropanoid–flavonoids pathways [96,97]. A high phenol level can effectively delay the growth of a pathogen at the infection site [95,98]; plants often increase their phenolic biosynthesis and antioxidant capacity after a pathogen infection [99,100]. Phenolic biosynthesis and/or antioxidant capacity in fruits can be induced by signaling molecules such as jasmonates and salicylic acids [101,102]. Our study, in line with the results of Mendoza et al. [101] and Wang et al. [95], showed that TSPC and TAC were increased by SA and MeJA treatments on the fruit inoculated with *M. laxa* compared to control treatments on the fruit inoculated with *M. laxa* (Figure 3).

A previous study by Zhu and Tian [103] demonstrated that JA increased fruit antioxidant capacity, which can result in a mediation of disease resistance of fruit, such as in this study (Figure 3). However, some fungi can hijack the JA signaling pathway and the pathogen can cause disease [104]. Balsells-Llauradó et al. [5] showed that *M. laxa* inoculation induced the early stage of JA biosynthesis, but a gene downregulation was detected in some cases of ripe fruit inoculated with *M. laxa* compared to control fruits [5]. The authors concluded that JA activity was likely useful as a plant defense signaling molecule [5]. This was in agreement with our results that MeJA increased the phenolic content, antioxidant capacity, and enzyme activities of fruits inoculated with *M. laxa* compared to control fruits (Figure 3 and Figure 4).

Plants naturally produce several plant enzymes, such as PAL, POD and SOD, which play roles in plant defense mechanisms (PDM) [105]. PAL has an essential role in the PDM because it catalyzes the biosynthesis of phenylpropanoids, which has a key role in the biosynthesis of phenols, phytoalexins, and lignins [48,104,105]. PAL activity in plants can be induced successfully when the plant organ is infected by microorganisms including fungal pathogens [34,35,105,106,107,108]. This was confirmed by our results; PAL activity increased in *M. laxa*-infected fruits when they were treated with MeJA and SA (Figure 4). This result with MeJA was in agreement with the studies of Yao and Tian [34,35], in which the authors demonstrated that MeJA induced a stronger PAL activity and decreased LD caused by *M. fructicola* or *P. expansum* in sweet cherry and peach fruits, respectively. This result indicates that SA and MeJA treatments on the *M. laxa*-inoculated fruits could induce resistance against *M. laxa*, because they activated the plant defense system by increasing phenol content after the infection (Figure 3), and this increase was accompanied with the enhanced activities of some enzymes (such as PAL and POD), which have a role in the biosynthesis of phenols (Figure 4).

Cell protection from oxidative stress was shown by SOD, an enzyme which can catalyze the dismutation of O^2−^ to oxygen and H_2_O_2_ [109,110]. POD catalyzes H_2_O_2_ to H_2_O and has a role in phenol oxidation during plant defense reactions [111,112]. Therefore, measuring SOD and POD activities gives indications on the amounts of reactive oxygen species (ROS), such as O^2−^ and H_2_O_2_. ROS are produced when fungal pathogens attack plant cell walls; therefore, increasing ROS is one of the first signs indicating a plant’s resistance to fungal pathogens [113,114]. Our results confirmed the above physiological process, because: (i) the temporal activities of both SOD and POD were significantly enhanced by SA and MeJA treatments in *M. laxa*-inoculated fruits (Figure 4); and (ii) activities of SOD and POD are well associated to each other in the plant defense system because a strong, positive relationship existed in the three inter-correlation analyses (Pearson correlation, regression analyses, and PCA) (Table 2 and Table 3, Figure 5 and Figure 6).

The plant cell wall has a connection to the activity of the POD enzyme, and this enzyme has a role in lignin biosynthesis [112]. Oxidation of peroxidases results in a more rigid cell wall through the increased matrix of polysaccharide and glycoprotein molecules [112,115]. Thus, POD plays a key role in the reinforcement of cell walls; therefore, it can protect the cell wall against infection by fungal pathogens [116,117,118,119]. Our results indicated that postharvest treatments of SA and MeJA induced higher POD activities in *M. laxa*-inoculated apricot fruits compared with water-treated fruits (Figure 4). The temporal increase in POD activities was in line with the reductions in BRI and LD (Figure 1A,B). This result with MeJA was in line with the studies of Yao and Tian [34,35] and Moosa et al. [119], in which the authors demonstrated that JA induced a stronger POD activity and decreased LD caused by *M. fructicola*, *P. expansum* and *P. digitatum* in sweet cherry, peach and citrus fruits, respectively.

Overall, the results on enzyme activities indicated that PAL, SOD and POD activities in MeJA and SA treatments were well associated to each other in the plant defense mechanisms against *M. laxa* in apricot fruit, which was confirmed by strong positive inter-correlations among the three enzymes (PAL vs. POD, PAL vs. SOD, and POD vs. SOD) in the *M. laxa*-inoculated fruit under MeJA and SA treatments (Table 2 and Table 3; Figure 5 and Figure 6).

## 5. Conclusions

In conclusion, MeJA and SA treatments played a vital role in promoting plant defense systems against a plant pathogenic fungus in the *M. laxa* vs. apricot pathosystem during SL storage conditions. The inhibition effects of SA and MeJA on brown rot (caused by *M. laxa*) on apricot fruit were as follows:

i. Direct reducing effect of disease incidence (BRI) and lesion diameter (LD) on infected fruits;
ii. Indirect reducing effect by increasing physical and antioxidant attributes (FF, LC, TAC and TSPC) of infected fruit, which resulted in an increase in fungal antagonistic compounds such as phenols and antioxidants in the infected fruits;
iii. Indirect reducing effect by inducing defense mechanisms in the infected fruit cells, which resulted in an increase in production and activity of defense-related enzymes such as PAL, POD and SOD; thus, the lignification of the cell wall was increased, which made the cell wall more mechanically rigid against fungal attack.

In summary, MeJA and SA are practically useful plant elicitors to enhance the plant defense system against brown rot by reducing fungal growth and by increasing physical and antioxidant attributes (FF, LC, TAC and TSPC) and the activity of defense-related enzymes (PAL, POD and SOD) during SL storage conditions.

## Figures and Tables

**Table 1:** The treatment effect of 0.1, 0.4 and 0.7 mmol L^−1^ methyl jasmonate (MeJA) and 0.5, 2 and 5 mmol L^−1^ salicylic acid (SA) on brown rot incidence (%, BRI) and on lesion diameter (mm, LD) of *Monilinia laxa* on cultivar ‘Bergarouge’ apricot fruit assessed at day 8 after the treatment began.

Treatments	Brown Rot Incidence (%)	Lesion Diameter (mm)
Water-treated control	58.42 ± 3.45 a ^1^	15.49 ± 1.01 a
MeJA		
0.1 mmol L^−1^	36.48 ± 1.96 b	15.77 ± 0.45 a
0.4 mmol L^−1^	22.84 ± 0.08 c	11.09 ± 0.69 c
0.7 mmol L^−1^	23.50 ± 0.72 c	12.81 ± 0.23 b
SA		
0.5 mmol L^−1^	32.35 ± 2.09 b	12.99 ± 0.59 b
2 mmol L^−1^	21.50 ± 2.12 c	10.31 ± 0.47 c
5 mmol L^−1^	22.50 ± 0.70 c	10.44 ± 0.61 c
LSD_0.05_	3.86	1.02

^1^ Different letters within the columns coupled with the BRI or LD values are significantly different at *p* = 0.05 according to LSD *t*-tests. Data are means ± SD from a triplicate assay.

**Table 2:** Pearson’s correlation coefficients (*r*) and their corresponding significance levels (*p*) amongst 7 fruit measures on apricot cultivar ‘Bergarouge’ for inoculated fruits with methyl jasmonate (0.4 mmol L^−1^) and salicylic acid treatments (2 mmol L^−1^). Data were combined for the assessment days 0, 2, 4, 6 and 8 in the SL storage period. Bold figures represent the significant (*p* < 0.05) correlation coefficient values.

Methyl Jasmonate (MeJA)						
	**FF**	**LC**	**TSPC**	**TAC**	**PAL**	**SOD**
LC	**0.885**					
	**0.011**					
TSPC	**0.800**	0.668				
	**0.046**	0.134				
TAC	**0.956**	**0.900**	0.668			
	**<0.001**	**0.005**	0.134			
PAL	**−0.838**	−0.736	−0.663	**−0.899**		
	**0.026**	0.079	0.139	**0.007**		
SOD	−0.698	−0.681	−0.453	**−0.817**	**0.909**	
	0.104	0.121	0.361	**0.041**	**0.003**	
POD	**−0.827**	−0.727	−0.553	**−0.911**	**0.952**	**0.936**
	**0.028**	0.085	0.294	**0.003**	**<0.001**	**0.001**
**Salicylic Acid (SA)**						
	**FF**	**LC**	**TSPC**	**TAC**	**PAL**	**SOD**
LC	0.783					
	0.061					
TSPC	**0.882**	−0.573				
	**0.006**	0.261				
TAC	**0.941**	**−0.867**	0.439			
	**<0.001**	**0.012**	0.384			
PAL	**−0.943**	**0.859**	−0.412	**−0.951**		
	**<0.001**	**0.016**	0.408	**<0.001**		
SOD	**−0.885**	0.765	−0.189	**−0.913**	**0.936**	
	**0.006**	0.071	0.727	**0.003**	**<0.001**	
POD	**−0.949**	**0.889**	−0.455	**−0.974**	**0.979**	**0.903**
	**<0.001**	**0.006**	0.358	**<0.001**	**<0.001**	**0.004**

Fruit parameters: FF, fruit firmness; LC, lignin content; TPC, total soluble phenol content; TAC, total antioxidant capacity; PAL, phenylalanine ammonia-lyase activity; SOD, superoxide dismutase activity; and POD, peroxidase activity. *n* = 25.

**Table 3:** Pearson’s correlation coefficients (*r*) and their corresponding significance levels (*P*) amongst 7 fruit measures on apricot cv. ‘Bergarouge’ for non-inoculated fruits with methyl jasmonate (0.4 mmol L^−1^) and salicylic acid treatments (2 mmol L^−1^). Further information on data use and bold letters are given in Table 2.

Methyl Jasmonate (MeJA)						
	**FF**	**LC**	**TSPC**	**TAC**	**PAL**	**SOD**
LC	0.598					
	0.232					
TSPC	**0.943**	−0.612				
	**<0.001**	0.204				
TAC	**0.896**	−0.714	**0.861**			
	**0.002**	0.092	**0.015**			
PAL	**−0.801**	0.705	−0.761	**−0.902**		
	**0.046**	0.101	0.070	**0.005**		
SOD	**−0.869**	0.716	**−0.826**	**0.927**	**0.971**	
	**0.015**	0.091	**0.029**	**<0.001**	**<0.001**	
POD	**−0.874**	0.695	−0.724	**−0.919**	**0.941**	**0.969**
	**0.013**	0.111	0.085	**<0.001**	**<0.001**	**<0.001**
**Salicylic Acid (SA)**						
	**FF**	**LC**	**TSPC**	**TAC**	**PAL**	**SOD**
LC	0.622					
	0.186					
TSPC	**0.849**	−0.614				
	**0.009**	0.202				
TAC	**0.897**	−0.671	**0.928**			
	**0.002**	0.132	**<0.001**			
PAL	−0.798	**0.863**	−0.776	**−0.841**		
	0.051	**0.015**	0.066	**0.022**		
SOD	−0.702	**0.878**	−0.621	−0.725	**0.944**	
	0.102	**0.012**	0.188	0.085	**<0.001**	
POD	−0.766	**0.931**	−0.689	−0.784	**0.962**	**0.975**
	0.071	**0.002**	0.116	0.061	**<0.001**	**<0.001**

Information is given in footnotes of Table 2.
